# Rhabdomyolysis-Induced Acute Kidney Injury (AKI) in a Young Bodybuilder: A Case Report

**DOI:** 10.7759/cureus.34625

**Published:** 2023-02-04

**Authors:** Jamsheer Parammal Alikutty, Anoop Raj, Sirajudheen K Soofi, Amer A Alkhateeb, Ahmed A Soliman, Fawwaz R Al Amiri, Ahmad A Abujaber, Mohamed Zuhail K Peediyakkal, Mohamad Khatib, Abdulqadir J Nashwan

**Affiliations:** 1 Critical Care Medicine, Hamad Medical Corporation, Doha, QAT; 2 Nursing, Hamad Medical Corporation, Doha, QAT

**Keywords:** nonsteroidal anti-inflammatory drugs, bodybuilding, ibuprofen, rhabdomyolysis, acute kidney injury

## Abstract

Acute kidney injury (AKI) is a serious medical condition that can have many causes, including rhabdomyolysis. Rhabdomyolysis is the breakdown of muscle tissue that can lead to the release of muscle fiber contents into the bloodstream. This can cause serious damage to the kidneys, leading to AKI. In this case, a young bodybuilder was diagnosed with rhabdomyolysis induced by AKI after consuming Ibuprofen for a casual fever. The etiology of AKI in rhabdomyolysis is complex, with multiple factors contributing to the development of the condition. These include muscle trauma, dehydration, infection, and drug toxicity. In this case, Ibuprofen may have contributed to the development of AKI, as it can cause kidney damage when taken in large doses. Additionally, the bodybuilder's physical activity may have contributed to the development of rhabdomyolysis, as intense exercise can cause muscle damage. Treatment for AKI in rhabdomyolysis patients typically involves aggressive fluid resuscitation, electrolyte replacement, and dialysis if necessary. Additionally, the underlying cause of the rhabdomyolysis must be identified and treated. In this case, the patient should be monitored closely for any signs of kidney damage, and the Ibuprofen should be discontinued. In conclusion, this is a case of a relatively common presentation with uncommon circumstances. It is crucial to have a heightened understanding of the likelihood of AKI in patients with rhabdomyolysis and the impact of drug toxicity in exacerbating the condition. Early diagnosis and treatment are essential for the successful management of AKI.

## Introduction

Rhabdomyolysis is defined by the acute destruction of skeletal muscles, muscle fiber apoptosis, and Fragmentary muscle fiber restoration. This is followed by releasing muscle cell contents, including intracellular metabolites (urea, potassium, and phosphorus) and intracellular proteins such as myoglobin, creatine kinase, aspartate transaminase, and lactate dehydrogenase, into the blood circulation [1]. In accordance with earlier studies, acute kidney injury (AKI) affects 10%-50% of rhabdomyolysis patients [2].

AKI in rhabdomyolysis has a complex etiology. Some possible causes are direct tubular toxicity of myoglobin, vasoconstriction, formation of intra-tubular casts, and renal ischemia caused by low blood volume [1]. AKI is witnessed in 34%-85% of marathon runners and is a common finding among endurance runners who run long distances [1]. Numerous population-based studies have impliedly demonstrated relations between nonsteroidal anti-inflammatory drugs (NSAIDs) and AKI, with relative risks compared to non-users [3].

NSAIDs are used extensively worldwide to self-manage fever, inflammation, and pain associated with various disease conditions. However, inappropriate use of NSAIDs, on the other hand, can significantly damage the body's organs and systems. Consumer reports in 2017 state that 39.7% of American households purchased ibuprofen as an over the counter [2]. When NSAIDs are misused, the most common side effects are damage to the digestive system and AKI [3]. Most of Qatar's population are laborers, and many regularly purchase NSAIDs from neighborhood pharmacies to alleviate occupation-related musculoskeletal pain. On the other end of the spectrum, sedentary employees in Qatar, who have a back pain prevalence of 59.2%, likewise abuse NSAIDs to treat their lifestyle-related pain [3]. This report aims to provide a comprehensive account of the progression and management of rhabdomyolysis-induced AKI within the context of ibuprofen and the muscular activity-related regimen.

This article has been posted as a preprint on Authorea [4].

## Case presentation

A 22-year-old male patient was admitted to the emergency department with abdominal pain, concentrated urine, and reduced urine output. The patient does not have any past medical history. He had a history of fever, for which he took one ibuprofen tablet. Thereafter, he developed abdominal pain, concentrated urine, and decreased urine output. He went to a private hospital and was found to have high creatinine and was referred to our general hospital for continuity of care.

**Figure 1:**
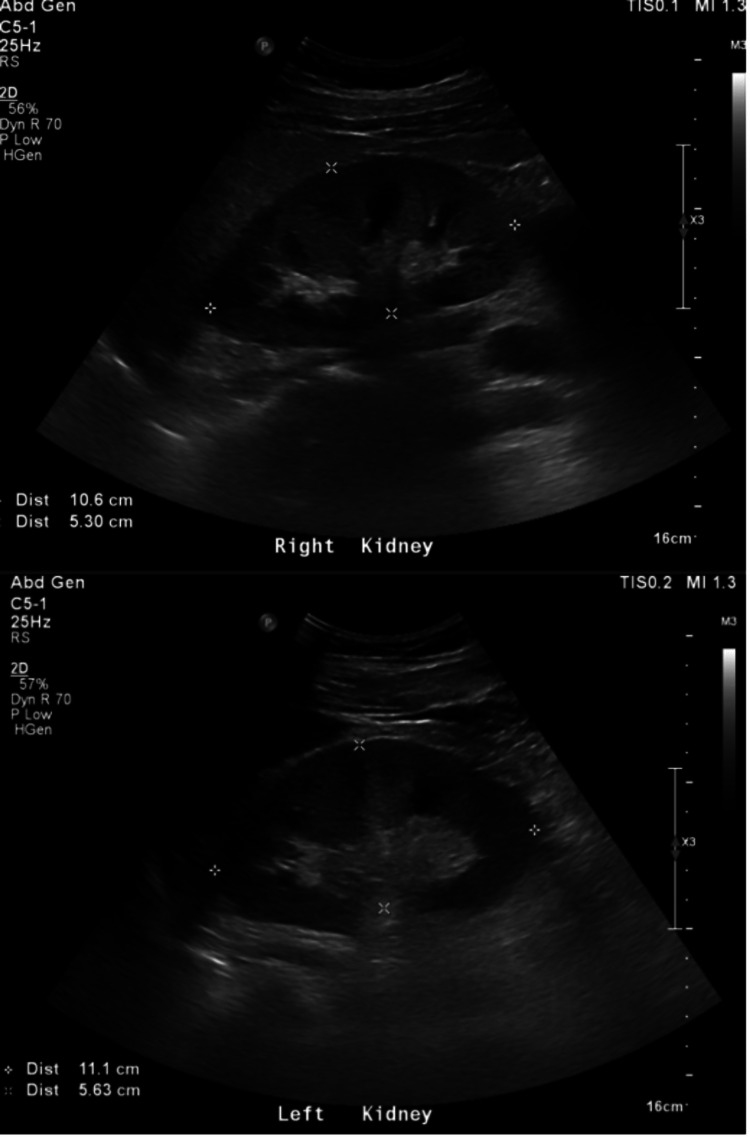
Ultrasound of the abdomen and pelvis shows both kidneys appear normal in shape, size, and echogenicity.

The patient was admitted to critical care with elevated kidney function, abdominal pain, and oliguria. Preliminary blood investigation showed high creatinine (517 µmol/L) and blood urea (13.6 mmol/L). While taking his history, he revealed that before his admission, he went to the gym for a workout, after which he developed muscle pain. An abdominal ultrasound (Figure 1) and CT-KUB were performed, and the study was unremarkable, apart from fatty liver. Results showed an increased value for creatinine kinase (CK) and myoglobin, which were > 22,000 µL and 1,902 ng/mL, respectively, which led to a diagnosis of AKI secondary to rhabdomyolysis.

**Figure 2:**
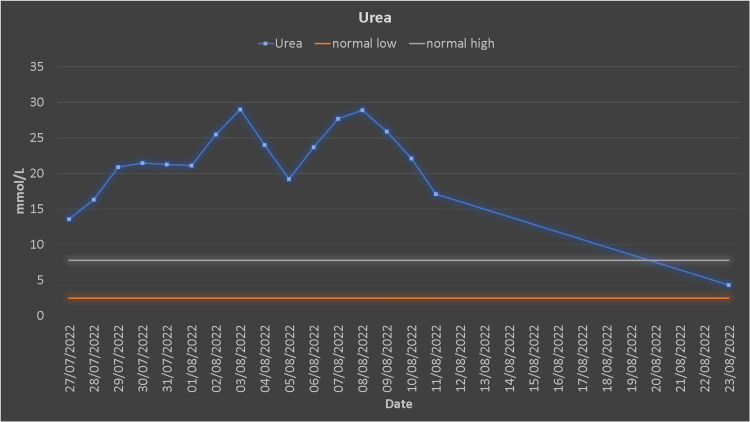
Urea trends.

**Figure 3:**
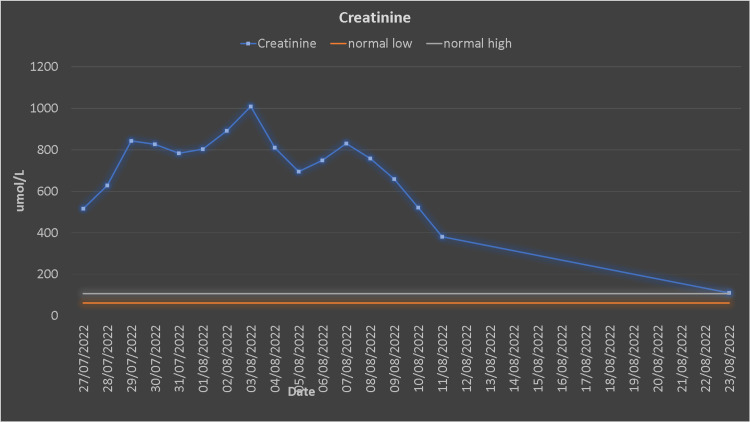
Creatinine trends.

The patient underwent urgent dialysis. After the first dialysis session, his creatinine, urea, CK, and myoglobin decreased (Figures 2, 3).

**Figure 4:**
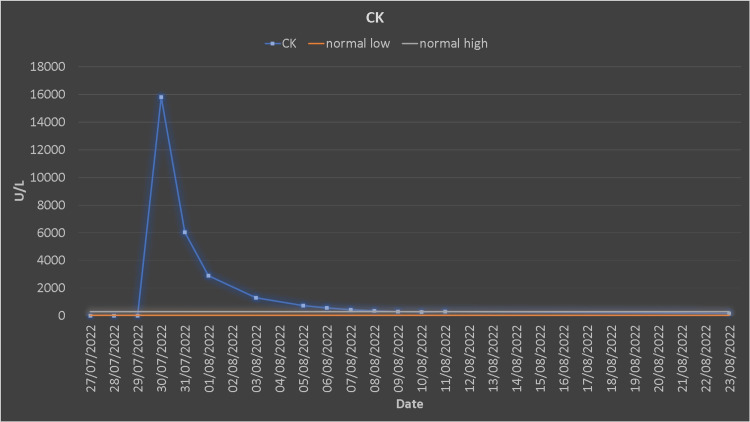
Creatine kinase trends.

**Figure 5:**
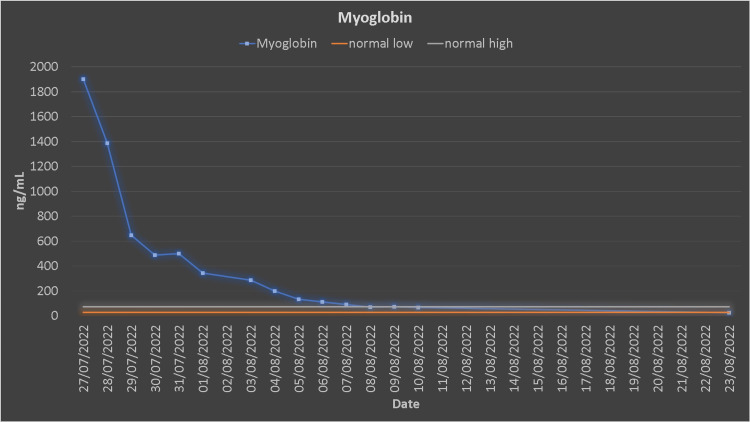
Myoglobin trends.

He was newly diagnosed as hypertensive, which was treated with antihypertensive medications. The white blood count (WBC) count was elevated (12.2 x 10^3^/µL), and he was febrile and had an increased body temperature (37.8 °C). Blood, urine culture, C-reactive protein (CRP), and procalcitonin were sent to the lab, and antibiotics were started simultaneously. After seven dialysis sessions, his myoglobin, creatine kinase (CK), urea, and creatinine reached the normal range (Figures 4, 5). The patient was discharged after 14 days of hospitalization.

## Discussion

The patient presented an unusual manifestation of rhabdomyolysis-induced AKI injury using ibuprofen and strenuous physical exercise. Ibuprofen is a NSAID drug formulated as tablets in 200 mg and 400 mg strengths with a half-life of 1.8-2 hours. It is one of the safest medicines used to treat fever, pain, and inflammation [5], with the most common side effects being gastric irritation, stomach ulceration, kidney injury, and skin reactions [5].

Rhabdomyolysis is usually caused by a sudden injury to a muscle leading to an influx of myoglobin leaking into the bloodstream, causing pain, weakness, tightness, and swelling in the muscles [6]. This may result in a series of physiological pathologies characterized by hyperkalemia, hyperphosphatemia, hyperuricemia, increased CK, and myoglobin influencing the stability of the internal environment, resulting in AKI due to myoglobin and toxins that obstruct renal tubules requiring intensive care unit management [7]. Rhabdomyolysis is associated with several illnesses and conditions. A variety of factors, including trauma to the muscle, intense physical activity or exercise, certain medications, and certain medical conditions, such as electrolyte imbalances, genetic disorders, and infections, can cause rhabdomyolysis [8]. In some cases, the cause of rhabdomyolysis is unknown. Risk factors for the condition include muscle trauma, crush injuries, heat stroke, severe burns, and alcohol or drug use. [8]. The pathophysiology of rhabdomyolysis-induced AKI is mediated by a toxin known as myoglobin, which is present in vertebrate muscles with greater oxygen retention than hemoglobin and aids myocytes in obtaining energy. It can be identified in urine at 5 g/L. Myoglobin-induced kidney toxicity is often characterized by intratubular cast formation, vasoconstriction, and direct toxicity to renal tubular cells [9].

Like other disorders, rhabdomyolysis may be better understood by detailed history collection and physical assessment. A comprehensive history is necessary to discover the cause of a muscle injury, including any family or personal history, trauma or exercise, and drug and/or illegal substance use before developing symptoms. In this case, the patient verbalized taking a single dose of high-risk medication (Ibuprofen) with a history of vigorous exercise, which is believed to be the triggering factor for developing rhabdomyolysis. However, laboratory serum CK tests were required to confirm the rhabdomyolysis diagnosis.

Typically, serum CK increases within 12 hr of the initiation of muscle damage, peaks between 24 and 72 hours, and returns to normal approximately five days after muscle injury [10]. Other investigations include serum myoglobin, potassium, calcium, urea, creatine, transaminases, lactate dehydrogenase, and urine dipstick [11].

The first step in managing rhabdomyolysis is treating the underlying cause giving equal importance to preventing AKI and treating metabolic issues that may come along with it, as both are known to complicate patient prognosis. Hemodialysis may be necessary to manage severe metabolic problems and control volume overload in around 85% of oliguric AKI patients and 30% of non-oliguric AKI. Initially, daily hemodialysis or continuous renal replacement therapy (CRRT) may be required to remove excess potassium and urea generated by damaged muscles [12,13]. However, on a positive note, published case reports and retrospective studies show that rhabdomyolysis can improve if treated quickly and aggressively [14,15].

Patients suffering from rhabdomyolysis should be educated on the risk factors and how to prevent them in the future. Education should cover the importance of preventing fluid imbalances associated with rigorous activity and precaution against NSAIDs [16,17].

## Conclusions

This is a case report of a young bodybuilder who developed symptoms after taking Ibuprofen for a casual fever. He was eventually diagnosed with AKI-induced rhabdomyolysis. This is an example of a relatively common presentation with unusual circumstances. In addition to educating patients on the risk factors and how to prevent them from happening again, healthcare providers should also emphasize the importance of monitoring for symptoms of rhabdomyolysis, such as muscle pain, weakness, and dark urine. Patients should be encouraged to seek medical attention if they experience these symptoms, as early diagnosis and treatment can help prevent further complications. Furthermore, patients should be advised to take medications as prescribed and to avoid taking more than the recommended dose. Finally, patients should be encouraged to stay hydrated and avoid strenuous activity, especially when taking medications. By following these guidelines, patients can reduce their risk of developing rhabdomyolysis and other complications.
